# System of Sensors and Actuators for the Production of Water Used in the Manufacture of Medicines

**DOI:** 10.3390/s19204488

**Published:** 2019-10-16

**Authors:** Fabricio Roosevelt Melo da Silva, Diego Antonio de Moura Fonsêca, Werbet Luiz Almeida da Silva, Elmer Rolando Llanos Villarreal, German Alberto Echaiz Espinoza, Andrés Ortiz Salazar

**Affiliations:** 1 Department of Computer Engineering and Automation, Federal University of Rio Grande do Norte (DCA-UFRN), Natal 59072-970, Brazil; fabricio@dca.ufrn.br (F.R.M.d.S.); diegomoura@dca.ufrn.br (D.A.d.M.F.); werbethluizz@hotmail.com (W.L.A.d.S.); andres@dca.ufrn.br (A.O.S.); 2 Department of Natural Sciences, Mathematics, and Statistics, Federal Rural University of Semi-arid (DCME-UFERSA), Mossoró 59625-900, Brazil; 3 Automation and Control Department, School of Electronical Engineering, National University of San Agustín de Arequipa (UNSA), Arequipa 04002, Peru; gechaiz@unsa.edu.pe

**Keywords:** automation, conductivity, MODBUS, purified water, sensors, SCADA, medicines manufacturing

## Abstract

This paper presents the development and implementation of a centralized industrial network for an automatic purified water production system used in the pharmaceutical industry. This implementation is part of a project to adapt an industrial plant to cope with advances in industrial technology to achieve the level of Industry 4.0. The adequacy of the instruments and the interconnection of the controllers made it possible to monitor the process steps by transforming a manual plant, with discontinuous production into an automated plant, improving the efficiency and quality of the produced water. The development of a supervisory system provides the operator with a panoramic view of the process, informing in real-time the behavior of the variables in the process steps, as well as storing data, event history and alarms. This system also prevented the collection of erroneous or manipulated data, making the process more transparent and reliable. Accordingly, we have been able to tailor this water treatment plant to operate within the minimum requirements required by the regulator.

## 1. Introduction

Water is one of the major commodities used by the pharmaceutical industry [1], and is the raw material most widely used in the manufacture of pharmaceutical products [2].

Water has unique chemical properties due to its polarity and hydrogen bonds. This means it can dissolve, absorb, adsorb, or suspend many different compounds, including contaminants [3].

Thus, it needs to be of high purity, and it is of great importance that its composition analysis is carried out to identify contaminants that may interfere with its quality, thus compromising the efficacy and quality of the produced medicines [2].

In the pharmaceutical industry, water can be used as a reagent, ingredient, or vehicle. However, there are formulas in which water is part of the composition of the product, such as injectables, eye drops, ophthalmic solutions, syrups, and suspensions, making it essential that the quality meets the specifications of use [4,5].

In this sense, water quality control plays an important role, as it is an essential tool that ensures the production of medicines meeting required specifications [6]. Therefore, the quality of the water used in the industry should follow standards according to its application [2]. According to [7], water for pharmaceutical use is classified into three categories: purified water (PW), Highly purified water (HPW) and water for injection (WFI), as shown in Table 1.

Given the importance of water in the drug production process, the Agência Nacional de Vigilância Sanitária (ANVISA) (“National Sanitary Vigilance Agency”) the regulatory agency responsible for medicines manufactured/marketed in Brazil, has determined rules and standards for Good Manufacturing Practices (GMP) for water for pharmaceutical use. These are found in [8] and [7], which set out, respectively, Good Manufacturing Practices and water quality requirements for Pharmaceuticals.

A water treatment system for pharmaceutical purposes consists of a pretreatment unit, treatment unit, water storage and distribution structure, process monitoring, and control devices and chemical cleaning and sanitation systems. Associated with these segments, the validation and qualification of water purification, storage, and distribution systems are a fundamental part of GMP and form an integral part of the GMP inspection [3,9].

Validation and qualification of water purification, storage, and distribution systems, as well as continuous production and a computerized system for monitoring and storing system data, are fundamental parts of GMP and are the requirements inspected by the regulatory agency. The latter being recently inserted, as the standards are updated continuously.

The Water Treatment Station (WTS) of the Núcleo de Pesquisa em Alimentos e Medicamentos (NUPLAM) (“Food and Drug Research Center”), located at the Federal University of Rio Grande do Norte, needed to changes, because it had treatment phases isolated from each other, with a totally discontinuous production, necessity of the present operator constant, and has no type of data storage system.

Given this problem, the main objective was to elaborate a project to adapt and update this treatment plant following the standards required by ANVISA. The project was divided into two stages. The first step (the focus of this article) is the implementation of an industrial network to establish a link among controllers, sensors, and actuators as a solution to enhance the connectivity of the legacy Water Treatment Station. This stage also contemplates the development of a SCADA (Supervisory Control and Data Acquisition) system with the particularity required by ANVISA reaching the minimum requirements for this WTS to operate within the legislation. However, the proposed project should consider adequacy only with existing equipment. This has become a challenge due to equipment from different manufacturers.

The second stage, expected to be implemented in another phase of the project, is the implementation of the paradigms that underlie Industry 4.0, such as the internet of things and data storage in clouds, as well as upgrading or replacing equipment (controllers, sensors, and actuators) that meets these characteristics. Therefore, in this last step, all these devices will be interconnected in a virtual repository where all information about each production data will be stored. New services derived from analyzing this stored data will be added, preparing the industry to enter Industry 4.0 or IIoT (Industrial Internet of Things) [10,11].

Using the latest software, we will be able to identify trends and patterns for specific processes and devise strategies to increase industry performance and efficiency throughout the production cycle. As well as provide feedback through corrective actions in the functional system that has remote access and control through advanced terminals and WebService outside the factory environment [12,13].

This article has been divided into five sections. In the first section, we had a brief introduction showing the importance of water in the pharmaceutical industry, the steps of this entire project, and the focus of our work on adapting a water treatment plant through automation. In Section 2, we will show the operation of the purified water treatment station, object of study, with its steps, functions, equipment, and characteristics. In Section 3, we show the proposed system with the implementations and modifications made in the plant. In Section 4, we will show some results and case studies on atypical days (maintenance or sanitization days) at WTS after the deployed system. Finally, in Section 5, we will make a brief conclusion about this work and future challenges of this project.

## 2. Materials and Methods

Before approaching the proposed automation solution to meet the requirements imposed by ANVISA, it is necessary to include, as a starting point, a general description of the Water Treatment Station (WTS). The WTS is the NUPLAM sector responsible for producing purified water that will be used in other factory sectors that need this product. Nowadays, the produced purified water is being used for cleaning and sanitizing materials, instruments, and surfaces, although it has the quality to be used in synthesizing drugs, in formulations and production of medicines.

In the process of producing purified water, the combination of two or more techniques to remove all types of contaminants is required [14]. Any purification methodology is permissible provided that the quality requirements for use are achieved. Purified water is commonly produced by ion exchange, reverse osmosis, ultrafiltration and/or electro-deionization processes and distillation [3]. Each of the purification technologies must be used in an appropriate sequence to optimize their particular removal capabilities.

A typical purified water system consists of various stages, depending on the methodology. In the WTS, four units compose the plant: pretreatment, treatment, water storage, distribution and loop structure, and chemical cleaning and sanitation systems. The treatment unit is subdivided into two stages: deionization and reverse osmosis. All units have automation elements such as metering pumps, sensors, actuators, controllers, etc. The tasks of each unit are now explained:

The schematic below shows the water purification system at WTS designed to produce purified water.

### 2.1. Pretreatment

The first step is pretreatment equipment specifically designed to remove contaminants in the feed water. Pretreatment removes contaminants that may affect purification equipment located downstream, especially reverse osmosis (RO) systems [15].

**Figure 1:**
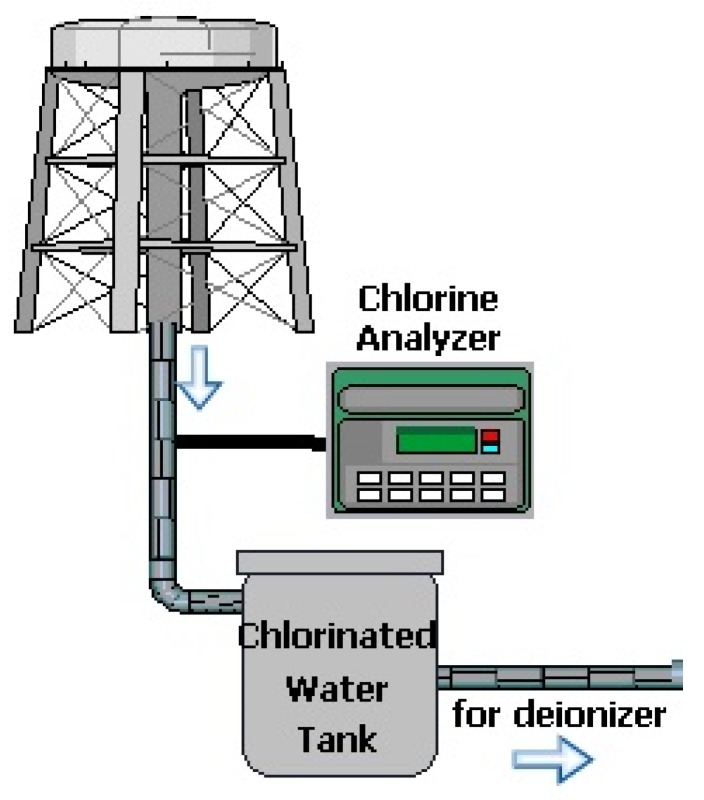
Pre-treatment system.

Pretreatment includes the addition of chlorine to control the growth of bacteria in pipes and tanks and a stage that incorporates sand filtration, which helps solids settle to the bottom of a storage tank. The first stage of the process starts before the water reaches WTS, with chlorine injection through a metering pump, still in the water tank, outside the factory (Figure 1).

Chlorinated water flows to the WTS through a 2″ PVC line. At the WTS, this water passes through a chlorine sensor that sends a 4–20 mA signal to the chlorine analyzer, which according to the established reference value (2 ppm), sends the command to the metering pump to inject or no chlorine in the water. Chlorinated water remains temporarily stored in a closed 500 L tank before being transferred to the next step, the deionization. The main instruments involved in the pretreatment step are the chlorine sensor, the HMI (Human-Machine Interface) chlorine analyzer/controller and the chlorine metering pump. The most relevant information to be measured at this stage is the amount of chlorine present in the water. Figure 1 illustrates this first stage.

### 2.2. Deionizer

The deionization process consists of the removal of cations and anions that are dissolved in water [16] by synthetic ionic resins in series. Initially, the cationic-exchange resin is followed by anionic-exchange, generating the replacement of ions by cations and anions present as impurities [17]. The cation-exchange resin captures ions such as calcium, magnesium, and sodium, resulting in an uptake of cations by the spatially fixed resin and release of hydrogen ions (H${}^{+}$) in exchange. In the anion-exchange resin, as anions are acquired by the anion-exchange, hydroxyl ions (OH${}^{-}$) are liberated. The cationic and anionic resins are regenerated with acids and bases, respectively [7]. This technique alone does not produce high purity water; for this reason, it is widely used in water pretreatment together with other processes, as it is effective in removing dissolved ions [8]. This process eliminates ions and non-organic matter, so it is easily contaminated [18,19].

At the WTS, the deionization process begins when chlorinated water stored in the tank is transferred to the deionizer system through a centrifugal pump. At the outlet tank, a metering pump injects a sodium metabisulfite solution in order to eliminate residual chlorine present in water, as it is a strong reducing agent used for removing chlorine and chloramines from water [7]. The metabisulfite injection is regulated by an ORP analyzer (indirect measure) that acts according to the ORP (Oxidation Reduction Potencial) sensor installed inline. The residual chlorine is eliminated to avoid the saturation of ionic resins prematurely.

**Figure 2:**
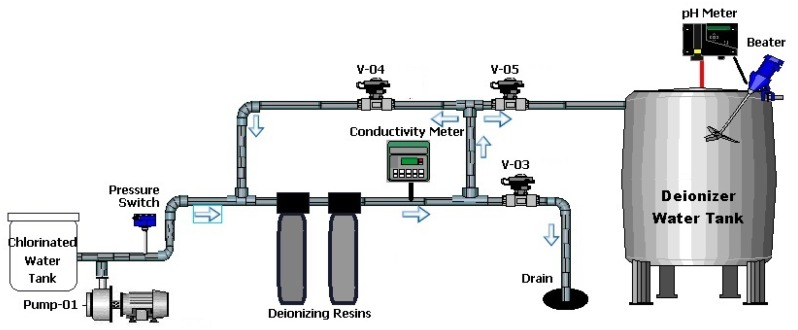
Deionization system.

The process begins by opening the V-03 valve for four minutes to discard water present in the piping and deionizer system. At the end of this period, the V-03 valve is closed, and the V-04 valve opens so that the water coming from the pretreatment circulates through the deionizer system for 6 min. Both periods can be modified by the operator via the PLC (Programmable Logic Controller) HMI. At the end of this time, the conductivity sensor installed at the output of the second (cationic) resin verifies the conductivity of the water. At this point, the water conductivity should begin to decrease below the setpoint value of 25 μS/cm, configured on the conductivity analyzer. If the conductivity value does not decrease as expected, it is indicative that the resins are saturated and ionic exchanges cannot be carried out. In this situation, the deionization system automatically begins the resin regeneration process, which consists of replacing the resin particles, cations, and anions uptake during normal operation with H${}^{+}$ and OH${}^{-}$ ions, respectively. Once the regeneration process is completed, the system begins a new deionization cycle, this time ensuring ionic exchange during the passage of water through the resins. When the water conductivity reaches the setpoint value, the conductivity analyzer sends a signal to the PLC to close valve V-04 and to open the valve V-05. This action releases the flow of water to the deionized water tank, which will serve as a buffer tank to supply the next stage of the process, reverse osmosis. The storage tank has a capacity of 2000 L and is made of 316 L stainless steel (Figure 2).

A Delta PLC (model DVP-PS02) controls, through dedicated software, the deionization process by detecting the conductivity and opening/closing valves. The whole process can be easily managed through an HMI panel on the side. In this deionization step, the primary variable analyzed is water conductivity.

### 2.3. Reverse Osmosis

Reverse osmosis (RO) is a representative demineralization process for which a semipermeable membrane is adopted to remove the dissolved substances from a solution. Liquid and only the marginal portion of some ions can pass to the permeate side through a semipermeable membrane, but the majority of the dissolved materials are rejected [20]. Reverse osmosis is the primary method used in the final treatment stage for the production of purified water [8].

**Figure 3:**
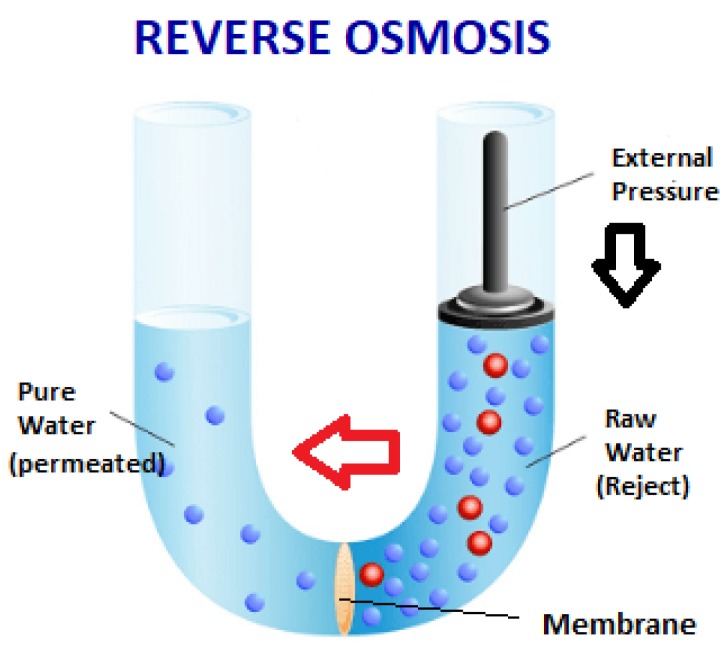
Reverse Osmosis.

RO membranes push water molecules from saline water to the less saline area by applying a hydraulic pressure greater than the osmotic pressure across a semipermeable membrane [21]. This semipermeable membrane inhibits the majority of dissolved impurities from passing through to the pure water side (Figure 3). The number of impurities carried over depends on the type and condition of the membrane (i.e., age and cleanliness) and the amount of pressure applied (energy) to the process [22].

The RO system produces one purified water stream called permeate and a second stream called concentrate, brine, or reject [22,23]. The permeable membranes permit the desalinated product water while inhibiting the passage of dissolved salts to pass through [24,25].

Due to the aromatic chain that makes up the membrane, the rejection rate of contaminants ranges from 90 to 99% [20,26,27,28].

In some RO system, two-stage membrane (or double passes) configuration is adopted for better solute rejection [29]. In the double pass reverse osmosis, the water produced by the 1st membrane group feeds a 2nd membrane group, duplicating the purification process. The 2nd group rejects are returned entirely to the storage tank.

Water from the deionizer system can contain chlorides and volatile organic compounds. Because these contaminants have a smaller physical size than water, the semi-permeable membranes used in the process are not able to retain them [8]. Membranes should, therefore, be adequately controlled as to deposition/scaling of calcium, magnesium and other salts, and biofilm, a critical source of microbial and endotoxin contamination [7,26]. In addition, different factors can significantly influence the separation of particles of impurities from water, such as pH, membrane differential pressure, and temperature. These factors may compromise process efficiency if there are no control methods for the water pretreatment that feeds the system [7].

A pH sensor inside the tank can provide a feedback signal to a metering pump inject acid or basic reagent and to a portable mixer, also installed in the tank, in order to homogenize the solution, and therefore, to maintain the pH within acceptable limits. Some types of RO membranes are sensitive to feed water pH and can become damaged if the pH is outside the recommended range of 5–8 pH.

Once the pH condition has been achieved, the water flows through a chiller heat exchanger to lower its temperature in order to achieve both criteria:

- Water conductivity is directly proportional to temperature. Therefore, the lower the temperature, the lower its conductivity.
- Reverse osmosis membranes have higher efficiency in feedwater below 20 °C (Increased feedwater temperature also results in lower salt rejection or higher salt passage).

**Figure 4:**
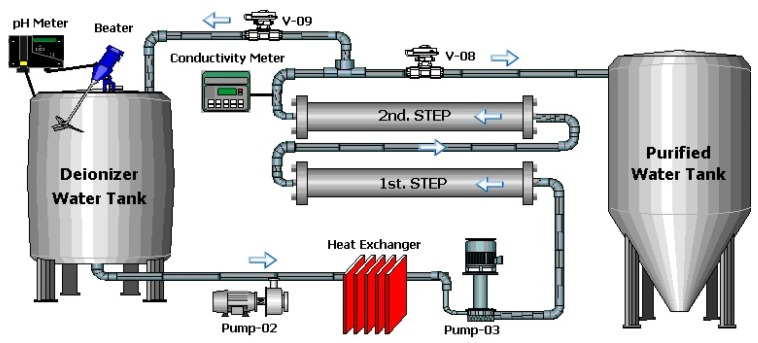
Reverse osmosis system.

The RO high-pressure centrifugal pump is typically multistage that continuously feeds the membrane system. Within the membrane system, the feedwater is divided into two streams according to the conductivity value indicated by the sensor installed at the second pass outlet, as illustrated in Figure 4. The low conductivity stream, called permeate, flows to the purified water tank through valve V-08 in case the conductivity value is lower than the setpoint value (0.7 μS/cm). The high conductivity flux, called concentrate, which values are greater than 0.7 μS/cm, returns to the deionized water tank for further processing. PLC controls the RO process by receiving information from the conductivity analyzer at the second pass outlet and commands the opening and closing of these valves (V-08 and V-07) according to the setpoint. System protection against high pressure or low pressures it is ensured by four pressure switches distributed throughout the reverse osmosis system.

### 2.4. Storage, Distribution and Loop

The storage and distribution system should be considered as a key part of the whole system and should be designed to be fully integrated with the water purification components of the system [3]. Once the water has been purified, it can either be used directly or, more frequently, it will be fed into a storage vessel for subsequent distribution to points of use [3]. The purified water distribution system is responsible for supplying all manufacturing areas that need water directly or indirectly, either in the manufacture or cleaning of the equipment used for this purpose [1,8,30].

The distribution of PW, HPW, and WFI should be accomplished using a continuously circulating pipework loop. The proliferation of contaminants within the storage tank and distribution loop should be controlled [1]. It should be subjected to a combination of online and off-line monitoring to ensure that the appropriate water specification is maintained [3]. Good justification for using a non-recirculating one-way system should be provided [1].

The PW water previously stored in the tank must supply the factory daily consumption points through a sanitary centrifugal pump. The PW storage should be designed to provide adequate protection, therefore, avoiding the water recontamination after treatment [8]. The higher the water purification quality, the faster the water tends to be contaminated in [7].

The distribution loop must be sized to meet the daily and peak usage demands for purified water during the reverse osmosis process. Maintainance of continuous turbulent flow circulation within the water distribution system reduces microbial contamination and biofilm formation [8,31].

The WTS’s storage, distribution, and loop system is composed by: a 316 L stainless steel insulated vessel (PW tank) with capacity of 2000 L, a heat exchange for bringing down the temperature (and reduce the risk of microbial growth), a circulation pump (with sanitary design and appropriate seals that prevent contamination of the system) to deliver purified water to a 316 L stainless steel circulation loop. A UV (ultraviolet) lamp is installed at the beginning of the loop to reduce the microbial load. A TOC (Total Organic Carbon) meter is installed at the return of the loop It measures both conductivity and Total Organic Carbon [32]. A temperature and level sensors monitor in real-time the current water temperature and PW tank level.

The centrifugal pump is applied in sending the water stored in the PW tank to a pure water circulation system, i.e., a closed-loop circulation system that uses a dedicated water pipe, with 11 consumption points, which loops throughout the factory, ending either at the pure water tank or the buffer tank. However, before return to the PW or buffer tank, the not consumed water passes through an inline TOC meter to measure the level of Total Organic Carbon. The heat exchanger reduces the circulating pure water temperature to around 19 °C.

**Figure 5:**
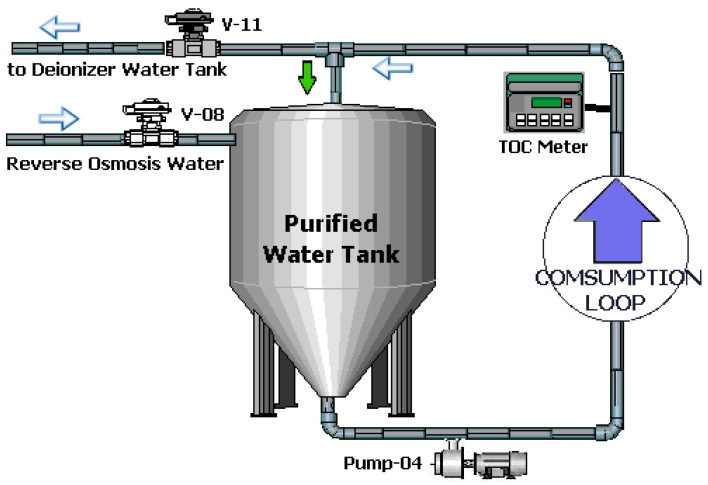
System: storage, distribution and loop.

Figure 5 presents the idea of the process of storage, internal distribution and return of water through the loop.

The recycle valve (V-11), which is an on/off valve, installed in the return line to buffer tank controls the volume of water that returns to the PW tank according to its level measured by a radar level sensor. Therefore, if the PW tank exceeds the pre-set maximum level, the water flows to the buffer tank. At the moment that the PW tank level reaches half of the maximum set point level, the PLC sends a command to close the valve (V-11). So, the level will rise again until it up to reaches the maximum level, starting then a new cycle.

The Storage, distribution, and loop process is controlled by a Siemens S7-1200 PLC. A Siemens TP-700 Confort HMI associated with the PLC provides information such as temperature, TOC, level in the storage tank and conductivity.

However, although the water purification process plant consists of the four required steps for the treatment, some adjustments must be made regarding the process in order to reach the new standards imposed by ANVISA.

## 3. Proposed Architecture

Considering the need to adapt WTS to ANVISA standards, improvements have been implemented, such as integrating the automation of each stage through an industrial serial digital communication network and the development of a supervisory system [33]. As already mentioned in the introduction, this initial implementation will make WTS meet the minimum operating requirements within current legislation.

**Figure 6:**
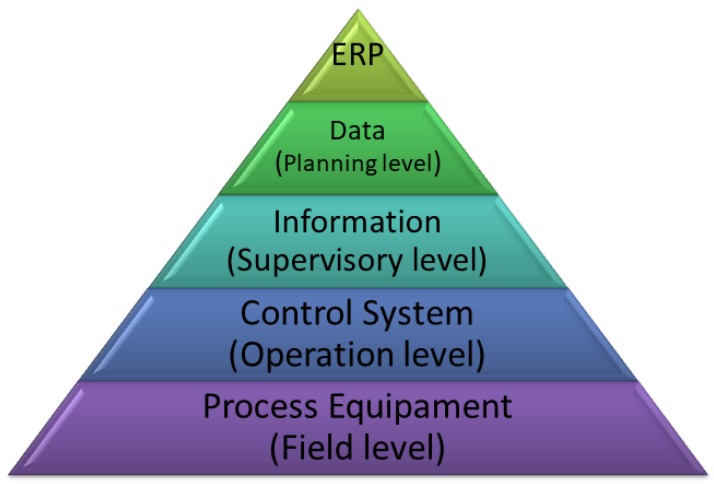
Automation pyramid.

Thus, a new hardware and software architecture based on Fieldbus class architectures is proposed to establish communication between the steps of the purified water production process. This proposal allows for dealing with the interoperability and integration of the controllers, sensors, and instruments involved in the automation system. The proposed architecture comprises the first three levels of the automation pyramid. Figure 6 (automation pyramid) illustrates this three-level topology implemented for vertical integration.

At the lowest layer are the sensors and actuators responsible for data acquisition, monitoring and alarm emission, actuation, and information provision at the top level. These instruments are connected directly to the controllers and, when endowed with intelligence, are integrated into the bus to which the controllers are also connected.

In the next layer or field layer, the main command unit is the PLC. These computational units are responsible for process control, processing and data provision to operators and gathering information about unit production, use of raw materials, energy and inputs and transfer to the higher level [34,35].

In the third tier, which covers supervisory, control, and monitoring system functions, the SCADA system is responsible for performing some functions in a manufacturing environment. Among these functions, we can highlight: operator communication with the various process steps, collection, storage and transfer of logical and analogical information about the system state, and can also generate control signals so that the controlled variable behaves according to presets setpoints.

In the future (at the end of the entire project) field devices and the cloud can communicate directly [36,37]. Thus, the new industrial communication hierarchy can be considered flat: services are available to any automation system participant [38,39]. For [40] and [41], one of the crucial points of industrial automation is communication, where there are a multitude of industrial communication systems adapted to data exchange, especially in the lower layers of the system.

All steps described in Section 3 except pretreatment include a PLC for performing tasks. A computer located in the control room gives the operator/user access to the supervisory application, from which it is possible to monitor and adjust process step parameters.

The integration between the PLCs of each process step, actuators and sensors were done through the MODBUS/RTU protocol in a standard RS-485 network that has a master-slave architecture [42]. The choice of this type of communication occurred because most of the equipment and instruments have in their structure the interface for this communication protocol. Instruments that did not have this type of communication, such as the chlorine analyzer, were connected to the analog inputs of the master PLC. The PLC chosen as the network master was the last step (storage, distribution, and loop) because it has more processing capacity, inputs, and outputs available. It also has two types of communication interfaces, Profinet (Ethernet standard) and RS-485, conditioning it to interconnect data between slave PLCs, peripheral instruments and SCADA. Table 2 shows the leading equipment present in the station, with its main functions and characteristics.

**Figure 7:**
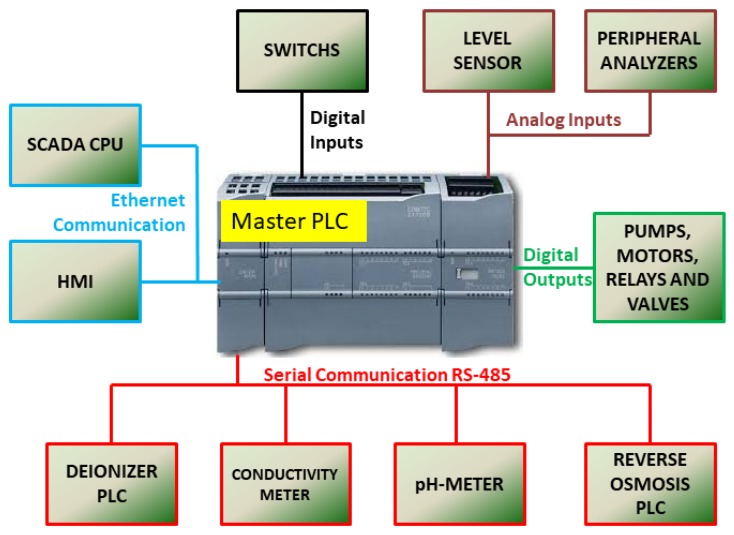
Automation system after interconnection of equipment.

For the communication between Elipse SCADA and the PLC, the application manufacturer has developed a communication driver, called Driver M-Prot; it allows communication using ISO/TCP protocol, or ISO over TCP [43]. This protocol is message-oriented, that is, it informs the length and end of the message, unlike TCP that has a deterministic network, it is data flow-oriented, where the message recipient is not aware of the size and where the message ends [44]. Thus, the distribution of the industrial communication network of the plant in WTS, shown in Figure 7, was as follows:

- Digital inputs and outputs: from the master PLC to inputs and outputs that use Boolean digital logic (0 and 1) or simply the signal on and off, such as buttons, relays, LEDs, lights indicators, etc.
- Analog inputs: between the master PLC and sensors and instruments that do not have RS-485 communication and use current signals (4 to 20 mA);
- Ethernet communication standard: it makes a connection between the master PLC, the HMI and the computer(PC) where the SCADA is installed;
- MODBUS communication (RS-485 standard): from master PLC to slave PLCs and equipment with RS-485 communication interface [45];

The master PLC is programmed through its manufacturer’s software. This PLC previously configured only with the control logic of the distribution, storage and loop step of the plant is now also responsible for centralizing the automation network. New commands have been implemented in their programming logic, adding new functions blocks responsible for the communication of the master PLC with the slave equipment. New data blocks have also been added, these for new addressing and storing data from slaves in the master PLC [46].

**Figure 8:**
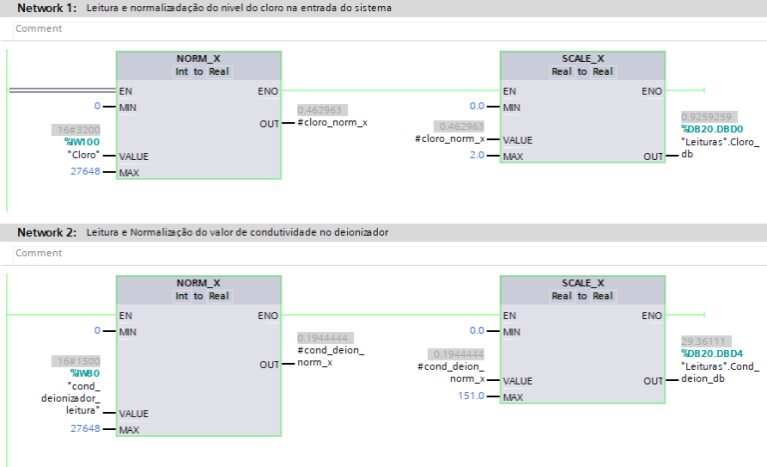
Configuration of the TIA Portal software to include the block diagram for normalization of the reading values of the variables.

The digital inputs and outputs of the PLC use voltage signals to define the boolean levels (0 and 24 Vdc), to represent levels 0 and 1, respectively. The analog inputs connected to the instruments receive a current signal, in the PLC this signal is converted into a term of 16 bits (word), which varies from 0 to 27,648, where 0 represents the lowest level of 4 mA, while 27,648 is the highest level of 20 mA [46]. Then for the master PLC to demonstrate the correct value read by the sensor, Is used the normalization and scheduling technique in its programming, according to Figure 8.

**Figure 9:**
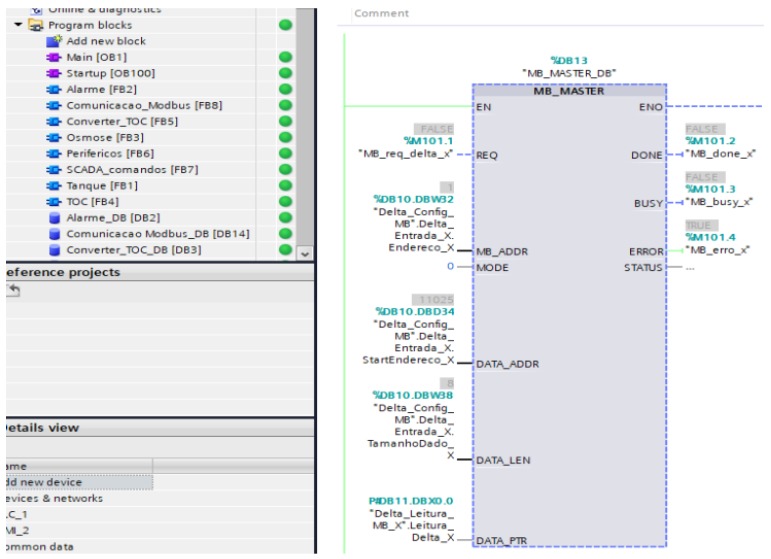
TIA Portal software configuration to add the block diagram of the slave equipment configuration in the new automation network.

According to the master/slave architecture of the MODBUS protocol, it was done a sequencing in the PLC configuration application. Thus, the master writes or reads in a slave, waits for his answer (one second at most, otherwise he jumps to the next in the queue), and continues the sequence, by reading ou write on to the next slave. This sequencing is cyclic, that is, after the response of the last configured slave returns to the first, and a new cycle begins. It is essential to configure three basic items (Figure 9), for correct reading the slave data in the master PLC: the slave address (MB_ADDR), the initial data memory address (DATA_ADDR) and the word length (DATA_LEN) [47].

The addressing (MB_ADDR) was done sequentially, from slave one (PLC Deionizer) to four (pHmetro). The initial address of the data memory (DATA_ADDR) changes from slave to slave and depends on the type of equipment. In the case of analysis and measurement equipment, they generally use a certain memory position fixed to express reading values. In PLCs, the amount of data is much greater: inputs, outputs, memory locations, internal keys, timers, among others. First, we verify the data that will be used in the SCADA, collecting them and configuring only those that are indispensable to the supervisor’s needs.

The word length (DATA_LEN) will be the number of memory locations to be read or written from the starting address, just as in the initial address they have fixed numbers, a boolean, a byte, an integer, etc., and can vary according to the need [47]. For example, to read the status of input X0 of slave 1. So MB_ADDR = 1 (Deionizer System PLC), this PLC has specific addresses for each type of variable to be collected, ie, there is one memory address (DATA_ADDR) for the inputs, another for the outputs, and so on. To read only the status of the input X0, set DATA_ADDR = 11,025 and DATA_LEN = 1. If it needs to read all physical inputs, from X0 to X7, the DATA_ADDR will be the same (11,025) and change the DATA_LEN to eight positions:(X0,X1,X2,…,X7). Another important configuration parameter in the function block is the DATA_PTR, which serves as a pointer to a block of data or bit memory from which data is to be written or read. This data is stored in a data block (DB) in the master PLC, and this DB block is available for reading or writing.

For the correct interaction between the PLC master and SCADA, the M-Prot driver is required to be installed and configured; it uses the ISO/TCP protocol to communicate with the PLC master [43]. The main parameters to be configured in the driver are a local address, rack, and slot where the PLC, it is physically located, the type of communication (Ethernet) and its IP address. After these settings, TAGs, are added, in the SCADA, and then their connectivity is tested with the PLC. All TAGs used in SCADA must also, be configured in the driver. This configuration will be the main point of connection between the PLC data and SCADA [48]. For a successful connection, four fields need to be configured: N1, N2, N3, and N4, which have the following characteristics:

1. N1 is the address of the PLC; there is only one PLC in the automation network communicating with SCADA, then N1 = 1 [43].
2. N2 is the reported data type; In this case, the data provided by the application manufacturer [46] is used, these values are multiplied by 100. For example, if N2 = 206, a byte data (2 × 100 = 200) is accessed at the digital inputs (200 + 6 = 206) [43].
3. N3 is filled only if the area to be read or written is in a datablock (DB) of the PLC; otherwise, it can be zeroed (N3 = 0) [43].
4. N4 is the offset address of the chosen DB; it will only be filled if a DB is used in the previous item (N3); otherwise, N4 = 0. For example, if N3 = 3 and N4 = 4, it means that we will be reading or writing one data in offset 4 of the DB3 of the PLC [43].

### SCADA

**Figure 10:**
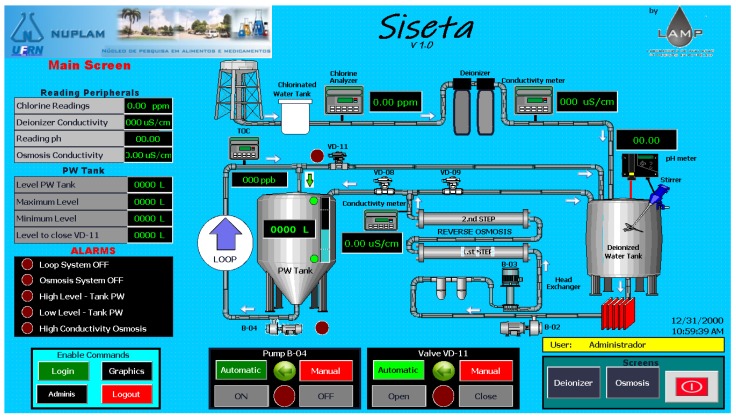
Main Screen of the Supervisory System (SISETA).

The developed system has features for generation and visualization of reports, necessary mainly during the validation process of the system. The Water Treatment Station Supervisory System (called SISETA, Sistema Supervisório da Estação de Tratamento de Água) also has user administration, event signaling, reporting and alarms feature [48]. For better interactivity with the user, this SCADA was developed in three different screens: the main screen, deionizer system, and reverse osmosis system, each one with its peculiarities. Figure 10 shows the first screen, called the main screen, where the user has a broad view of the WTS structure with the central monitoring and control parameters. On this screen there are the readings of the PW tank variables and setpoints, the main plant measuring instruments, the system status, if the loop pump is turned on or off, if the loop recycling valve is open or closed, besides the alarms.

**Figure 11:**
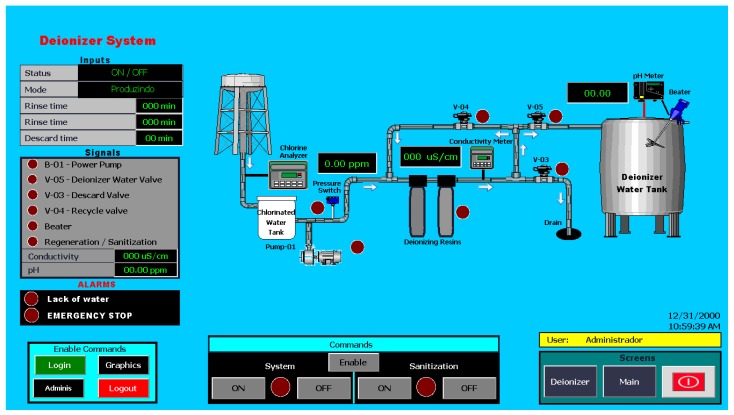
Deionizer System Screen (SISETA).

Figure 11 shows the second screen, called Deionizer, showing the step of the same name, showing the state and the times of the internal processes, the readings of the measuring instruments of this stage, besides the alarms. As in the first screen, the reading variables can be seen in the side panel and the plant illustration.

**Figure 12:**
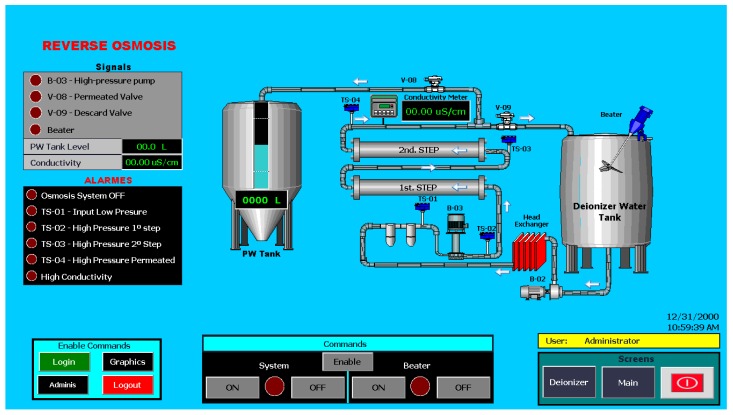
Screen of reverse osmosis system (SISETA).

Figure 12 shows the third screen, reverse osmosis, with a view of the PW tank level, the conductivity of this stage, and its alarms. All screens have commands to enable and disable their systems, but they will only be accessible if the logged-in user has permission to do so; otherwise, they will only be able to view the screens and will not be able to execute any commands. On all screens, there are viewing and printing commands, which allow you to access the daily reports of the main variables of the system.

There are other technical requirements to be met by a SCADA system for the production of purified water in the pharmaceutical industry in Brazil, the main ones (implemented in SISETA) listed in Table 3. By way of comparison, the requirements commonly implemented in a supervisory system for a water and sewage treatment plant were listed.

## 4. Results and Discussions

**Figure 13:**
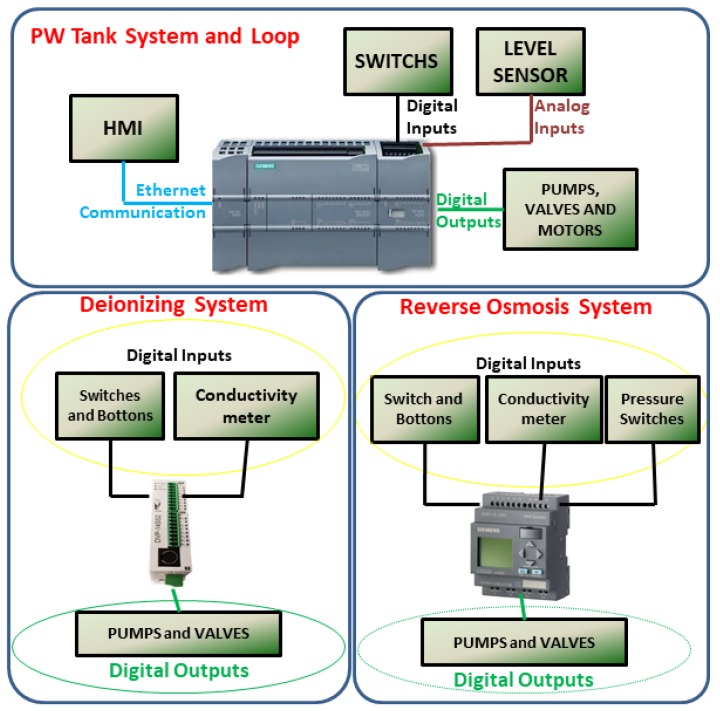
Decentralized systems before intervention.

The integration of water production phases into a single PLC facilitated the collection of variable data. Figure 13 illustrates the architecture of the plant before the intervention, totally decentralized, and without any communication between the stages of production. However, after the intervention, with the PLC of the last stage becoming the master and brain of this new architecture, according to as seen in Figure 7, we have a unified and centralized system.

After deploying SCADA in conjunction with this new network architecture, users can monitor and control the WTS of the supervisory room, managing, and interacting with the process, making it an important process tool. The system assists the operators in any intervention, achieving an increase in the efficiency and reliability of the process operation. The values of the variables correspond to those read in the instruments in real-time, and the reports are being generated and archived daily in a safe place, avoiding manipulation of data. Concerning reports, the system generates and stores to disk on the supervisory PC.

**Figure 14:**
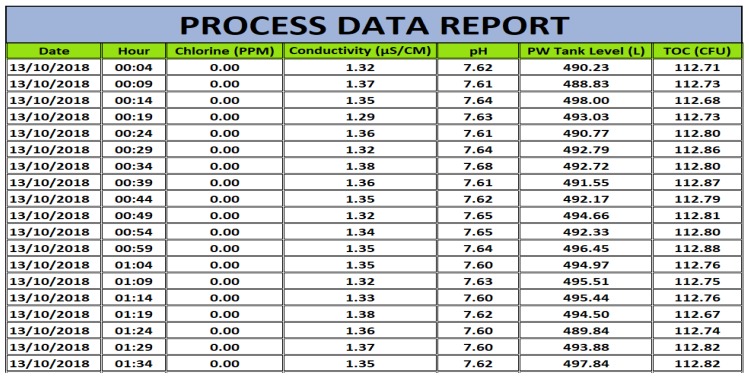
Daily report of the system stored in the SCADA PC.

Figure 14 illustrates part of a report generated. This report aims to prove the performance of the system over the days and, consequently, in the months in which it is in the validation period, imposed by ANVISA [7]. Before the implementation of the system, these data were collected manually, which gave rise to error or even data manipulation, thus having no legal value for validating these values to the regulator.

Therefore, with this data stored, it can, if necessary, create performance graphs and verify if there were any system abnormalities during the day, week, month, or year. It can verify anomalies for a specific period or even the behavior of the system after some operator procedure, such as cleaning and sanitizing the system.

**Figure 15:**
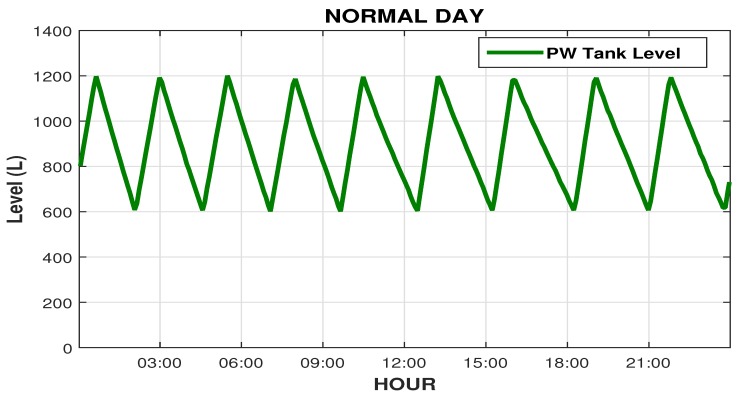
Sketch of the PW tank level reading graph.

Figure 15 illustrates the graphical of the PW tank level in its regular operation, you can see well the increase and decrease the water level in the PW tank, it is seen that the maximum setpoint it is set to 1200 L, and o the closing level of the recycle valve is 600 L, half the maximum setpoint defined to the operator.

To better exemplify supervision and control of the system by the automation network in conjunction with the sensors and the SCADA system, we chose atypical days of the system. These days are usually days when the system goes through some intervention and/or maintenance; then we made the graphs with the data stored.

**Figure 16:**
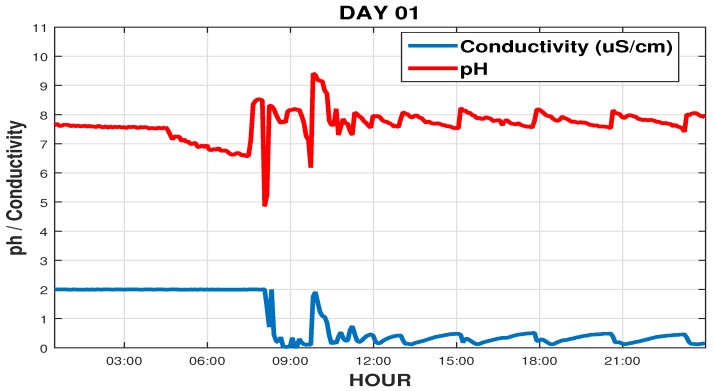
pH readings and RO conductivity during one day of intervention.

This maintenance occurred due to the significant increase in conductivity in the osmosis system output, as well as a significant variation in ph values, as shown in Figure 16, where these setpoints, its usually set at 0.7 μS/cm and 7.5, respectively. After brief maintenance on the system it is observed that around 12 hs A.M., the conductivity decreased and was below the setpoint, a sign that the sanitization of the RO had been satisfactory, but the pH (which this measured in the tank before RO) still had a wide variation, based on these data, operators see the need also to sanitize the deionizer system.

**Figure 17:**
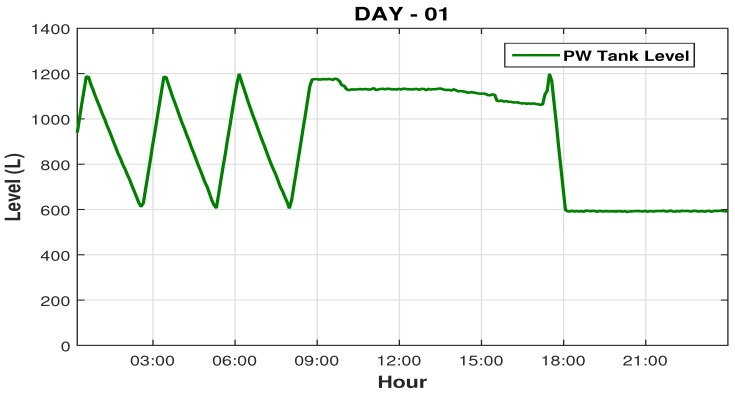
Sketch of graphical PW level reads during an intervention.

This second intervention takes a long time, about 6 hours long, so the loop system was interrupted for a long time outside its normal cycle, as can be seen in Figure 17, its noted that the PW tank level operates at normal cycle (ranging from 600 to 1200 L), until there was a shutdown of the system for maintenance and sanitization (cleaning) of the system (around 8:50 hs A.M.), we can observe that this maintenance continues until 5 hs P.M., the system restarts to lower the PW tank level, maintenance will continue the next day.

**Figure 18:**
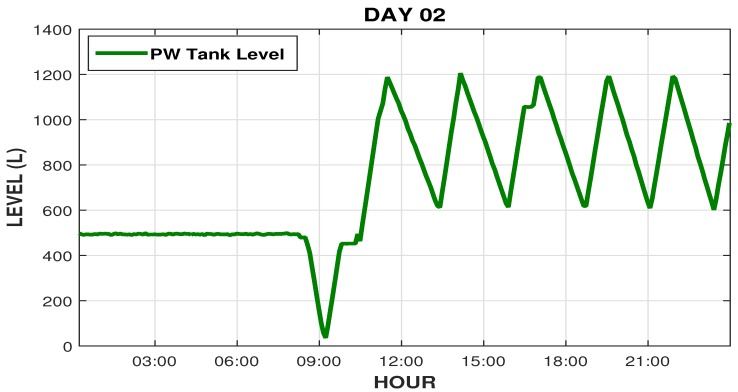
Readings of the PW tank level on the second intervention day.

On the following day (second day) operators continued system maintenance, Figure 18 shows the graphic outline of tank level behavior on the second day of the intervention. Note that the system was out of operation, the operators dried the entire tank to produce whole new water. So, around 10 A.M. (after maintenance) the distribution and loop system, was reactivated and the PW tank level returned to work in its normal cycle, ranging from 600 to 1200 L.

**Figure 19:**
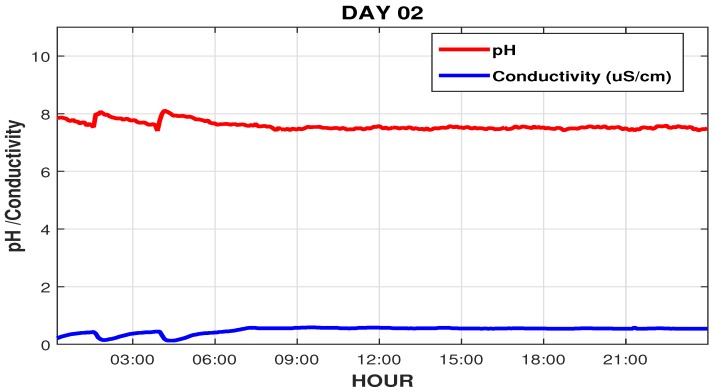
pH and RO conductivity readings on the second intervention day.

We can see that the conductivity, which before the intervention was 2 μS/cm, is now below the setpoint (0.7 μS/cm), and the pH that was varying above 8, now varying within the desired range near 7.5, as shown in Figure 19.

**Figure 20:**
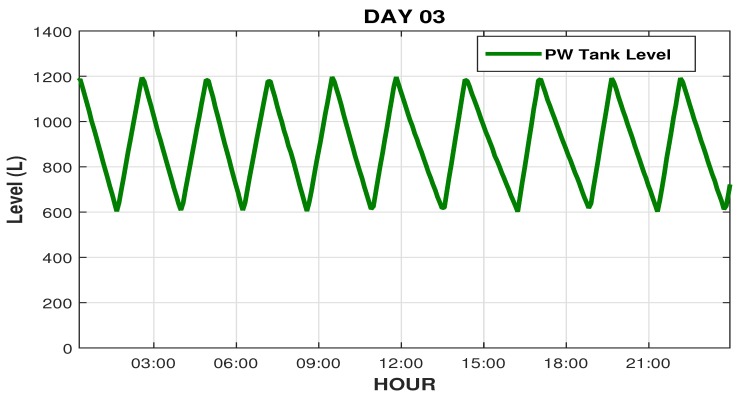
Readings of the PW tank level on the third day.

**Figure 21:**
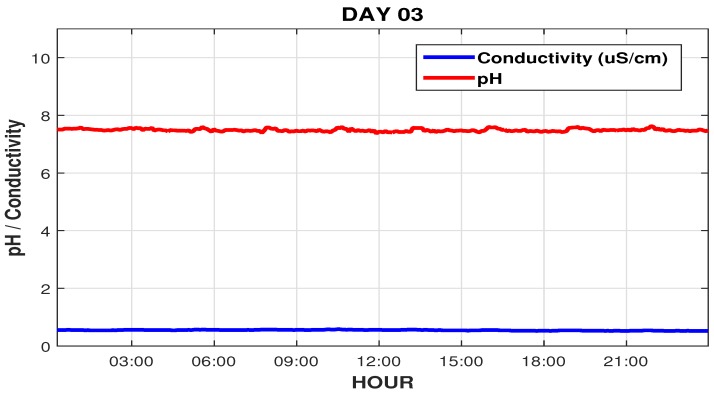
pH readings and RO conductivity on day 3.

Figure 20 illustrates the system behavior on the day following the interventions (third day), showing the regular rise and fall cycle of the PW tank (600 to 1200 L). Figure 21 shows the graphs of pH reading and osmosis conductivity graphs, respectively, which are within their reading and control ranges (0.7 μS/cm).

Thus, collecting this data in real-time greatly facilitated the diagnosis for operator intervention in the plant, made the system more transparent so that the supervisor can see if the procedures and interventions of the operators in the system are correct, as well as prove that the data collected matches the truth of the water validation process and is within the required standards.

## 5. Conclusions

This paper presented the implementation of a series of adjustments in the plant of the water treatment plant for pharmaceutical purposes concerning automation. The initial proposal consists of the implementation of a communication link between a controller, sensors and actuators network and a SCADA system as a solution to enhance the connectivity of the WTS. The most relevant contribution of the proposed solution is that all the operations of the WTS are performed automatically and autonomously. The developed SCADA system (SISETA) implements functionalities required by the Brazilian regulatory agency (ANVISA) such as audit trail, asset management, access control, electronic signature, etc. Besides, the presented system takes advantage of the already available components, regarding the implementation of the paradigms that govern the concept of industry 4.0.

The presented results prove the feasibility of the proposed solution for this first step of the project; that is, the information of the sensors, actuators, and controllers are effectively shared and managed through the Fieldbus, available for higher hierarchical level applications. This step will serve as the basis for the implementation of the Industry 4.0 concept. Future works include the addition of more industry 4.0 features like IoT and Cloud storage. Interoperability between instruments can be achieved through a data acquisition interface compatible with the sensor of the application field. Protocols like MODBUS TCP, that is an open de facto standard, and is used for some automation and control systems, and can be implemented as an IoT protocol. 

## Figures and Tables

**Table 1:** Types and main parameters of pure water.

Types of Water	Conductivity (μS/cm)	TOC(CFU)
Purified water	Up to 1.3	Up to 100
Water for Injectables	Up to 0.7	Up to 50
Highly Purified Water	Up to 0.1	Up to 10

**Table 2:** Leading Equipment.

Equipament	Location	Model	Main Features	Current Function
Delta PLC	Deionizer System	DVP-SS2	8-Digital Inputs, 6-Digital Outputs, Communication RS-232 and RS-485	Controls deionizer system logic
Logo PLC	RO System	Siemens-Logo	4-Digital Inputs, 8-Digital Outputs (with additional module)	Controls RO system
Siemens PLC	Loop System	S7-1200	8-Digital Inputs, 6-Digital Outputs, 6-Analog Inputs (2-Internal and 4-Auxiliary Module), Communication Ethernet and Communication RS-485 (Auxiliary Module)	Master PLC
HMI	Loop System	Siemens-TP700 Comfort	2-Communication Ethernet	Internal HMI in the Station
SCADA CPU	Supervisor’s Room	Desktop HP Intel Dual Core	Communication Ethernet	SCADA System
Peripheral Analyzers	In every plant	Miscellaneous Models	Communication RS-485 and use current signals (4 to 20 mA)	Analyser Chlorine, pH, Level Sensor, TOC, Conductivity meters

**Table 3:** Main requirements for the validation of the supervisory system (ANVISA).

SISETA	SCADA for Wastewater Treatment Plants
Controlled access to the supervisory application	Automation of emergency procedures
Automatic user logoff	Monitoring and control of the system
Versioning of the application with electronic signature	Graphic display of all informations
Presentation of encrypted data in a readable format	Diagnostic and security management
Redundant recording of electronic records	Alarm management, notification and optimization
Encryption of Electronic Data Records	Data elaboration in real time, trend and reports for offline data analisys
Audit Trail occurrence records	
Detection of improper alteration of electronic records	
Alarm configuration and control with notification	
Display of sensor values in real-time	

## Abbreviations

The following abbreviations are used in this manuscript:

WTS	Water treatment station
NUPLAM	Núcleo de Pesquisa em Alimentos e Medicamentos
PW	Purified water
HPW	Highly Purified Water
WFI	Water For Injection
GMP	Good Manufacturing Practices
SCADA	Supervisory Control and Data Acquisition
WFI	Water for injections
CFU	Chain forming unit
HMI	Human-Machine Interface
PLC	Programmable Logic Controller
TOC	Total organic carbon
RO	Reverse osmosis
ORP	Oxidation Reduction Potencial
PWT	Purified Water Tank
DB	Data block
ANVISA	Agência Nacional de Vigilancia Sanitaria
SISETA	Sistema Supervisorio da Estaçao de Tratamento de Água
